# Age-Related Variations in Lower Facial Soft-Tissue Thickness: A Retrospective Cephalometric Study

**DOI:** 10.7759/cureus.114314

**Published:** 2026-08-10

**Authors:** Mohit A Kathole, Tanuj Choudhari, Pradeep Kommi, Shirish Goel, Srijit Chattopadhyay, Deeksha Miryala

**Affiliations:** 1 Orthodontics and Dentofacial Orthopaedics, Maitri College of Dentistry and Research Centre, Durg, IND

**Keywords:** cephalometrics, facial soft tissue thickness, lip thickness, lower facial aesthetics, orthodontics

## Abstract

Background: Facial esthetics and orthodontic planning depend on both the craniofacial skeleton and overlying soft tissue. Lower facial soft-tissue thickness (FSTT) varies with sex, age, and ethnicity; however, age-stratified normative data remain limited for the Central Indian population. This study compared lower facial soft-tissue parameters across age groups and established age- and sex-specific reference values.

Materials and methods: A total of 120 left lateral cephalograms (60 male and 60 female) of subjects aged 8-39 years with normal vertical skeletal pattern (SN-MP = 32° ± 2°), complete dentition, and no orthodontic/surgical history were divided into four equal age groups (I: 8-15, II: 16-23, III: 24-31, and IV: 32-39 years; 15M/15F each). Six soft-tissue parameters-upper and lower lip thickness, upper lip length, Pogonion-Pogonion′ (Po-Po′), Menton-Menton′ (Me-Me′), and lower facial third height-were traced manually. One-way analysis of variance (ANOVA) with Tukey’s post hoc test was used to compare age groups, and independent t-tests were used to compare genders within groups (p < 0.05).

Results: Upper lip thickness increased significantly with age (F = 4.42, p = 0.006), peaking in the 32-39-year group; male subjects exhibited significantly greater upper lip thickness from eight to 31 years (independent-samples t-test, p < 0.05). Lower lip thickness (p = 0.19) and length of upper lip (p = 0.07) did not vary significantly with age. Po-Po′ rose to a peak in the 24-31-year group before declining (F = 3.91, p = 0.010). Me-Me′ increased progressively with age (F = 6.77, p = 0.01). The height of the lower facial third increased significantly with age (F = 4.63, p = 0.004), with a gender difference only in the youngest group (p = 0.05).

Conclusion: The lower facial soft tissue continues to change well beyond skeletal maturity. Upper lip, chin, and lower facial height measurements increased significantly with age, whereas lower lip thickness and upper lip length remained comparatively stable. Sexual dimorphism is most pronounced in adolescence/early adulthood and diminishes thereafter, supporting the need for age- and sex-specific soft-tissue norms in orthodontic and esthetic practice.

## Introduction

Facial morphology reflects both the skeletal framework and the overlying soft-tissue drape, the latter ultimately determining the facial contour [1]. Orthodontic planning has shifted from purely hard-tissue metrics to soft-tissue balance, as skeletal correction alone does not guarantee an esthetic profile. The lower facial third (lips, chin, and submental region) is central to attractiveness, lip competence, and functional harmony. Soft-tissue thickness varies with age, sex, ethnicity, and body composition; aging alters collagen, elastin, and fat distribution, producing lip flattening, deeper labiomental folds, and altered chin projection [2].

Lateral cephalometry [3] remains a cornerstone of orthodontic diagnosis. Classical analyses by Holdaway incorporated soft tissue as central to facial balance, establishing the “soft-tissue paradigm” [4]; Arnett et al. later formalized a comprehensive soft-tissue cephalometric analysis demonstrating marked sexual dimorphism [5]. Soft-tissue profiles are known to evolve through childhood and adolescence independent of skeletal growth [6,7] and continue remodeling into adulthood [8]. Population-specific studies from Lebanon, Sudan, and Iraq show facial soft-tissue thickness (FSTT) cannot be extrapolated from Caucasian norms [9-11], yet few studies have evaluated sequential age-group changes in lower FSTT using a standardized retrospective cephalometric protocol. Furthermore, facial soft-tissue evaluations must account for underlying vertical growth variations when establishing normative diagnostic values [12]. This study therefore aimed to determine and compare lower facial soft-tissue parameters across age groups and establish age- and gender-specific reference values.

In addition, orthodontic mechanics capable of modifying lower anterior facial height, including molar distalization, intrusion, and maxillary protraction, may indirectly influence soft-tissue profile and facial esthetics. Recent randomized clinical trials have demonstrated significant skeletal and dentoalveolar changes following these treatment modalities, emphasizing the importance of establishing reliable baseline soft-tissue norms before treatment planning [13-15].

## Materials and methods

Study design

A retrospective cross-sectional cephalometric study included 120 lateral cephalograms of individuals from Chhattisgarh, India, retrieved from the records of the Department of Orthodontics and Dentofacial Orthopaedics, Maitri College of Dentistry and Research Centre, Anjora, Durg, Chhattisgarh, India. The study was approved by the Institutional Scientific and Ethical Committee (Approval No. MCDRC/2024/APR/408; approved on April 2, 2024).

A total of 120 left lateral cephalograms (60 male and 60 female) were obtained from patients reporting to the Department of Orthodontics and Dentofacial Orthopaedics for treatment, using the Genoray Papaya 3D Plus system (Genoray Co., Ltd., Seoul, South Korea). Subjects aged 8-39 years with a normal vertical skeletal relationship (SN-MP angle = 32° ± 2°) to eliminate vertical skeletal variation as a confounding factor; complete dentition (with or without third molars); good-quality radiographs taken in natural head position; no history of previous orthodontic treatment, orthognathic surgery, or facial cosmetic procedures; clinically acceptable facial symmetry; and no craniofacial trauma, congenital anomalies, or systemic conditions affecting craniofacial growth were included. Subjects with poor-quality radiographs, craniofacial anomalies, prior surgical or orthodontic intervention, significant facial asymmetry, missing anterior teeth, or extreme body mass index (BMI) (outside WHO normal limits) were excluded.

The sample was divided into four age groups of 30 participants each (15 males and 15 females per group): Group I, 8-15 years; Group II, 16-23 years; Group III, 24-31 years; and Group IV, 32-39 years. The age groups were selected to represent different stages of craniofacial growth and maturation: active growth (8-15 years), completion of skeletal growth (16-23 years), stable young adulthood (24-31 years), and early adulthood, when age-related soft-tissue remodeling begins (32-39 years).

Radiographic technique

All cephalograms were obtained by the same operator under identical technical settings, with subjects in natural head position, seated condyles, teeth in centric occlusion, and lips relaxed. A midsagittal positioning beam ensured symmetric alignment, and a Frankfort horizontal (FH) positioning beam ensured correct anteroposterior head tilt. A film-to-focus distance of 152.4 cm and a film-to-median-plane distance of 10 cm were used for all exposures.

Tracing and landmark identification

**Figure 1:**
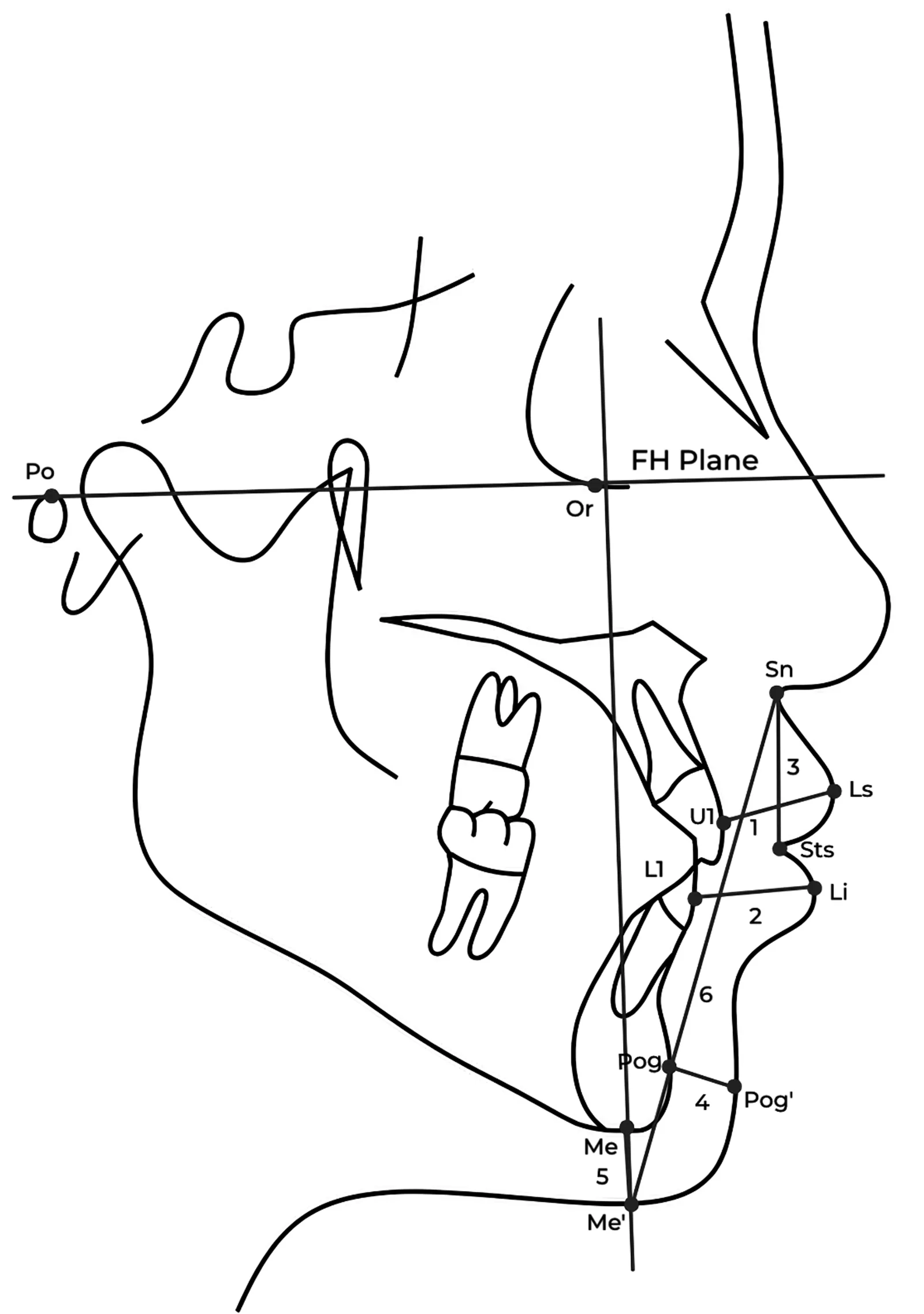
Cephalometric landmarks, reference plane, and linear soft-tissue measurements used in the study. The Frankfort horizontal (FH) plane was established by joining Porion (Po) and Orbitale (Or). Soft-tissue landmarks included Subnasale (Sn), Labrale Superius (Ls), Stomion Superius (Sts), Labrale Inferius (Li), Soft-Tissue Pogonion (Pog′), and Soft-Tissue Menton (Me′). Skeletal landmarks included the upper incisor (U1), lower incisor (L1), Pogonion (Pog), and Menton (Me). Linear measurements comprised (1) upper lip thickness (U1-Ls), (2) lower lip thickness (L1-Li), (3) upper lip length (Sn-Sts), (4) Pogonion-Pogonion′ thickness (Pog-Pog′), (5) Menton-Menton′ thickness (Me-Me′), and (6) lower facial third height (Sn-Me′). The figure was prepared (manually traced) by the authors specifically for this study based on the cephalometric landmarks and measurement protocol used in their research.

Cephalograms were manually traced on 0.003-inch lead-acetate tracing paper using a 4H pencil on an illuminated tracing table, with all landmarks defined according to Jacobson [16]. Where bilateral structures failed to superimpose, the average outline was used by inspection. Six soft-tissue parameters were recorded in millimeters: upper lip thickness (linear distance between the labial surface of the maxillary incisor and Labrale Superius (Ls), the most anterior point on the vermilion border of the upper lip); lower lip thickness (linear distance from the most prominent labial point of the mandibular incisor (L1) to Labrale Inferius (Li), the median point on the lower margin of the membranous lower lip); upper lip length (distance from soft-tissue Subnasale (Sn), the point where columella merges with the upper lip in the midsagittal plane, to Stomion Superius); Pogonion-Pogonion' (Po-Po') (linear distance between skeletal pogonion, the most anterior point of bony chin in the median plane, and soft-tissue pogonion, the most prominent point on soft-tissue contour of chin); Menton-Menton' (Me-Me') (linear distance between skeletal menton, the most inferior midline point on mandibular symphysis, and soft-tissue menton, the lowest point on contour of soft-tissue chin, located by dropping a perpendicular from the FH plane passing through skeletal menton to identify the corresponding soft-tissue menton (Me')); and lower facial third height (linear distance from soft-tissue Subnasale (Sn) to soft-tissue Menton (Me') as shown) (Figure 1).

To assess intra-observer reliability, 20 randomly selected lateral cephalograms (16.7% of the total sample) were retraced and remeasured by the same investigator after a two-week interval under identical conditions. The intraclass correlation coefficient (ICC) was calculated for all continuous cephalometric measurements, while Cohen's kappa coefficient was used to evaluate the reproducibility of landmark identification where applicable. The ICC values ranged from 0.89 to 0.96, indicating excellent agreement, and Cohen's kappa values ranged from 0.85 to 0.93, demonstrating almost perfect agreement according to the criteria proposed by Landis and Koch [17]. These findings confirmed excellent intra-observer reliability and reproducibility of the measurements.

Sample size calculation

The sample size was calculated using G*Power software (version 3.1; Heinrich-Heine-Universität Düsseldorf, Düsseldorf, Germany). An effect size (Cohen's d) of 0.74, two-tailed hypothesis, significance level (α) of 0.05, and statistical power (1 − β) of 90% were used for the calculation. The estimated minimum sample size was 22 participants per group. Considering four age groups, the minimum total sample size required was 88 participants. To enhance statistical power and improve precision of estimates, 120 participants were ultimately included in the study, with 30 participants in each age group.

Statistical analysis

Data were analyzed using descriptive statistics (sample size, minimum, maximum, mean, and standard deviation) for all parameters across age groups and genders. Statistical analyses were performed using IBM SPSS Statistics for Windows, Version 22.0 (released 2013; IBM Corp., Armonk, NY, USA). Prior to inferential statistical analysis, the distribution of all continuous variables was assessed for normality using the Shapiro-Wilk test. The test confirmed that the data were normally distributed (p > 0.05), thereby satisfying the assumption for the use of parametric statistical tests. One-way analysis of variance (ANOVA) was used to compare mean values among the four age groups for each of the six soft-tissue parameters, followed by Tukey's post hoc test for pairwise intra-group comparison where the overall ANOVA was significant. An independent-samples t-test was used for gender-wise comparisons within each age group. Age-wise comparisons were performed using one-way ANOVA. For all inferential analyses, the corresponding test statistic (F or t) and p-value were reported. Tukey's post hoc test was applied following significant ANOVA results. Statistical significance was set at p < 0.05.

## Results

A total of 120 subjects (60 male and 60 female subjects) were evenly distributed across four age groups of 30 participants each. Descriptive statistics for age and gender distribution are summarized in Table 1; standard deviation values were low across all subgroups, confirming homogeneity of age distribution within each group.

**Table 1:** Descriptive statistics for age and gender among study participants. Values are presented as mean ± standard deviation (SD). Each age group included 15 male and 15 female participants (total N = 120). N: number of participants

Age	Gender	Samples (N)	Mean age	SD
8-15 years	Male	15	12.06	1.75
	Female	15	12.00	2.00
16-23 years	Male	15	20.40	1.45
	Female	15	20.13	1.55
24-31 years	Male	15	27.13	1.18
	Female	15	27.93	1.70
32-39 years	Male	15	35.46	1.68
	Female	15	35.06	1.83

Age-wise comparison across parameters

Mean values, F-values, and p-values for all six parameters across age groups are summarized in Table 2.

**Table 2:** Age-group comparison of lower facial soft-tissue parameters (mean ± SD, mm). Age-wise comparison of lower facial soft-tissue parameters using one-way analysis of variance (ANOVA). Data are presented as mean ± standard deviation (SD). F-statistics and corresponding p-values are reported. Tukey's post hoc test was performed for pairwise comparisons when the overall ANOVA was significant.

Parameter (mm)	8-15 yrs	16-23 yrs	24-31 yrs	32-39 yrs	F-value	p-value
Upper lip thickness	11.50 ± 1.67	10.83 ± 1.95	11.05 ± 1.61	12.50 ± 2.37	4.42	0.006
Lower lip thickness	11.71 ± 1.43	12.00 ± 1.74	11.93 ± 1.41	12.56 ± 1.66	1.52	0.190
Upper lip length	16.52 ± 2.54	18.06 ± 2.47	17.76 ± 2.37	17.63 ± 2.16	2.38	0.070
Po-Po′	9.13 ± 1.90	9.93 ± 2.07	10.83 ± 2.16	9.35 ± 2.23	3.91	0.010
Me-Me′	5.85 ± 1.23	5.88 ± 1.56	6.63 ± 1.38	7.13 ± 0.98	6.77	0.010
Lower facial third height	58.06 ± 9.73	60.03 ± 4.53	63.05 ± 6.00	63.46 ± 4.12	4.63	0.004

One-way ANOVA demonstrated a significant difference in upper lip thickness among the four age groups (F = 4.42, p = 0.006), highest in the 32-39-year group and lowest at 16-23 years; post hoc differences were significant for 32-39 vs. 16-23 years (p = 0.006) and vs. 24-31 years (p = 0.022). One-way ANOVA showed no significant difference in lower lip thickness among the age groups (F = 1.52, p = 0.190). One-way ANOVA revealed no statistically significant difference in upper lip length across the age groups (F = 2.38, p = 0.070), rising from 16.52 mm to a peak of 18.06 mm at 16-23 years before stabilizing.

One-way ANOVA demonstrated a significant age-related difference in Po-Po′ (F = 3.91, p = 0.010), peaking at 24-31 years (10.83 ± 2.16 mm) before declining (p = 0.036 vs. 32-39 years). One-way ANOVA demonstrated a significant increase in Me-Me′ with advancing age (F = 6.77, p = 0.010), with the 32-39-year group differing from both the 8-15- and 16-23-year groups (p ≤ 0.002). One-way ANOVA demonstrated a significant increase in lower facial third height among the age groups (F = 4.63, p = 0.004), the 8-15-year group differing from the 24-31- and 32-39-year groups (p ≤ 0.018).

Gender-wise comparison across parameters

Male-female comparisons within each age group, across all six soft-tissue parameters, are summarized in Table 3. Males consistently exceeded females for upper lip thickness, upper lip length, Po-Po′, and Me-Me′ during adolescence and young adulthood, whereas lower lip thickness showed a gender difference only at 16-23 years and lower facial third height only at 8-15 years. For every parameter, gender differences were most prominent in the younger age groups (8-15 to 24-31 years) and were absent by 32-39 years, indicating a general convergence of male and female soft-tissue dimensions with advancing age.

**Table 3:** Gender-wise comparison (male vs. female, mean ± SD, mm) of lower facial soft-tissue parameters by age group. Values are presented as mean ± standard deviation (SD). Gender-wise comparisons of each lower facial soft-tissue parameter were performed within each age group using the independent-samples Student's t-test. The corresponding t-statistic and p-value are reported for each comparison. A p-value < 0.05 was considered statistically significant. Po-Po′: soft-tissue pogonion thickness; Me-Me′: soft-tissue menton thickness

Parameter	Age group	Male (mean ± SD)	Female (mean ± SD)	t-value	p-value
Upper lip thickness (mm)	8-15 yrs	12.53 ± 1.44	10.46 ± 1.18	4.20	0.001
	16-23 yrs	11.83 ± 1.70	9.83 ± 1.69	3.40	0.003
	24-31 yrs	12.00 ± 1.64	10.10 ± 0.84	3.90	0.001
	32-39 yrs	12.46 ± 2.16	12.53 ± 2.64	-0.076	0.94
Lower lip thickness (mm)	8-15 yrs	11.66 ± 1.49	11.76 ± 1.42	-0.18	0.89
	16-23 yrs	12.60 ± 1.72	11.40 ± 1.60	1.93	0.05
	24-31 yrs	12.30 ± 1.38	11.56 ± 1.38	1.44	0.15
	32-39 yrs	12.40 ± 2.02	12.73 ± 1.26	-0.54	0.59
Upper lip length (mm)	8-15 yrs	17.90 ± 1.96	15.13 ± 2.33	3.50	0.003
	16-23 yrs	19.43 ± 2.22	16.70 ± 1.93	3.50	0.001
	24-31 yrs	17.86 ± 2.64	17.65 ± 2.14	0.24	0.81
	32-39 yrs	17.63 ± 1.96	17.63 ± 2.42	0.00	1.00
Po-Po′ (mm)	8-15 yrs	9.33 ± 1.98	8.93 ± 1.86	0.56	0.88
	16-23 yrs	11.10 ± 1.28	8.76 ± 2.09	3.67	0.01
	24-31 yrs	11.53 ± 2.13	10.13 ± 2.02	1.84	0.07
	32-39 yrs	9.46 ± 2.66	9.23 ± 1.78	0.28	0.78
Me-Me′ (mm)	8-15 yrs	6.13 ± 1.49	5.56 ± 0.86	1.27	0.21
	16-23 yrs	6.53 ± 1.64	5.23 ± 1.20	2.47	0.02
	24-31 yrs	7.33 ± 1.11	5.93 ± 1.29	3.14	0.001
	32-39 yrs	6.83 ± 0.87	7.43 ± 1.01	-1.70	0.07
Lower facial third (mm)	8-15 yrs	61.50 ± 4.14	54.62 ± 12.40	2.30	0.05
	16-23 yrs	61.50 ± 3.70	58.56 ± 4.91	1.84	0.07
	24-31 yrs	62.93 ± 7.19	63.16 ± 4.77	-0.10	0.91
	32-39 yrs	63.66 ± 3.65	63.26 ± 4.66	0.26	0.79

The independent-samples t-test demonstrated significantly greater upper lip thickness in males than females at 8-15 years (t = 4.20, p = 0.001), 16-23 years (t = 3.40, p = 0.003), and 24-31 years (t = 3.90, p = 0.001), whereas no significant difference was observed at 32-39 years (t = -0.08, p = 0.94). The independent-samples t-test demonstrated a significant gender difference only in the 16-23-year group (t = 1.93, p = 0.05). The independent-samples t-test demonstrated significantly greater upper lip length in males at 8-15 years (t = 3.50, p = 0.003) and 16-23 years (t = 3.50, p = 0.001). The independent-samples t-test demonstrated a significant gender difference only in the 16-23-year group (t = 3.67, p = 0.01). The independent-samples t-test showed significantly greater Me-Me′ values in males at 16-23 years (t = 2.47, p = 0.02) and 24-31 years (t = 3.14, p = 0.001). The independent-samples t-test demonstrated a significant gender difference only in the youngest age group (t = 2.30, p = 0.05).

## Discussion

This study evaluated age- and gender-related variation in lower FSTT in a Central Indian population. Lower facial soft tissue continued to remodel well beyond skeletal maturity, with the magnitude and timing of change differing by landmark and sex, consistent with the soft-tissue paradigm of Holdaway and Arnett et al. [4,5]. Upper lip thickness increased progressively with age, agreeing with Kumar et al., who attributed continued change to soft-tissue remodeling, muscular tonicity, and fat redistribution [18]; comparable values in other Indian cohorts reinforce an ethnic influence on norms [19].

Lower lip thickness and upper lip length showed no significant age effect, suggesting earlier stabilization related to functional demands during speech and mastication [6], and consistent with Farkas et al. and Legan and Burstone, who described vertical lip growth as predominantly adolescent and skeletally governed [20,21]. Chin parameters showed distinct trajectories: Po-Po′ peaked in the mid-20s before declining, mirroring the biphasic pattern concluded by Farkas et al. and Sforza et al. [20,22] and Behrents’ observation of continued adult remodeling with later reduction [23], whereas Me-Me′ rose progressively, in keeping with Piombino et al.’s report of cumulative soft-tissue deposition with age [24]. Lower facial third height increased with age, corroborating Bishara et al. and Velemínská et al. on continued vertical facial growth into adulthood [25,26]. Understanding baseline soft-tissue dimensions is critical, as soft-tissue profile changes continue dynamically across developmental stages [27].

Sexual dimorphism was greatest in adolescence and young adulthood for upper lip thickness, upper lip length, Po-Po′, and Me-Me′, consistent with hormonal and muscle-mass differences [5], but diminished by the fourth decade for several parameters, reflecting convergence of soft-tissue dimensions with age [25]. Clinically, because thick soft tissue can mask skeletal/dental discrepancies while thin tissue accentuates them, decisions on incisor retraction, extraction, and camouflage versus surgical correction should be individualized by age and sex rather than generic adult norms [2,5]; continued chin and lower-facial remodeling into the fourth decade is also relevant to orthognathic planning and forensic facial approximation.

Recent cone-beam computed tomography (CBCT)-based evidence further supports that FSTT is influenced by age, sex, and skeletal pattern. Park et al. demonstrated significant differences in FSTT among skeletal Classes I, II, and III, with males exhibiting greater tissue thickness than females, highlighting the importance of individualized soft-tissue assessment during orthodontic treatment planning [28]. Similarly, Sadek and Alali reported that facial profile attractiveness is strongly influenced by lip thickness, lip protrusion, and ethnicity, concluding that universal cephalometric norms may not be applicable across different populations [29]. These findings support the use of population-specific soft-tissue norms, as established in the present study for the Central Indian population. Sultanova et al. also demonstrated that FSTT varies significantly with age, sex, and BMI, reinforcing that demographic factors should be considered when interpreting facial soft-tissue measurements and planning orthodontic treatment [30].

Limitations

As a two-dimensional cephalometric analysis, this study cannot capture three-dimensional (3D) or dynamic soft-tissue behavior, and midline measurements do not reflect bilateral variation. The single-institution, single-region sample may limit generalizability, and the cross-sectional design precludes true longitudinal tracking. Future studies using CBCT or 3D facial scanning, larger multi-centric samples, and longitudinal follow-up are warranted. BMI, nutritional status, and facial morphology were not analyzed and may influence FSTT.

## Conclusions

Lower FSTT varied significantly with age at several landmarks: upper lip thickness, Me-Me′, and lower facial third height increased progressively with age, Po-Po′ peaked in early adulthood before declining, and lower lip thickness and upper lip length remained relatively stable. Sexual dimorphism was most evident in adolescence and young adulthood and diminished by the fourth decade. These findings support the use of age- and gender-specific, rather than universal adult, soft-tissue reference values in orthodontic diagnosis, esthetic planning, and facial reconstruction in this population.
